# Commentary: Cytokine-Regulation of Na^+^-K^+^-Cl^−^ Cotransporter 1 and Cystic Fibrosis Transmembrane Conductance Regulator-Potential Role in Pulmonary Inflammation and Edema Formation

**DOI:** 10.3389/fimmu.2017.01490

**Published:** 2017-11-06

**Authors:** Michael Eisenhut

**Affiliations:** ^1^Paediatric Department, Luton and Dunstable University Hospital NHS Foundation Trust, Luton, United Kingdom

**Keywords:** cystic fibrosis transmembrane conductance regulator, Na^+^-K^+^-Cl^−^ cotransporter 1, sweat chloride, sweat sodium, nasal potential difference, pulmonary edema, sodium, chloride

In their minireview on cytokine-regulation of Na^+^-K^+^-Cl^−^ cotransporter 1 (NKCC1) and cystic fibrosis transmembrane conductance regulator (CFTR), the authors propose a model of the pathogenesis of inflammation induced pulmonary edema formation involving a combination of activation of these channel systems leading to alveolar fluid secretion and an inhibition of function of CFTR and epithelial sodium channel (ENaC) depending on localization of the channel system in small airways (1). The authors have not taken into account data obtained from *in vivo* measurements of ion transport correlates in patients with inflammation-related pulmonary edema. In children with meningococcal septicemia-associated pulmonary edema ventilated on a pediatric intensive care unit, we detected increased sweat and salivary chloride and sodium levels in patients with septicemia-related pulmonary edema. Respiratory epithelial chloride channel but not sodium channel function was reduced as evident from nasal potential difference measurements (2). The model proposed by the authors Weidenfeld and Kuebler needs to consider these data and the fact that the ratio of sodium to chloride in sweat and saliva was always greater than one in our investigation, which is against a channel system dysfunction affecting primarily the chloride transport as proposed. The authors propose a combination of combined CFTR and ENaC inhibition and a CFTR and NKCC1 activation and possible ENaC inhibition depending on localisation of channel systems within the airway epithelium, which would both together result in a net greater increase in chloride in the apical epithelial lining fluid compared to sodium because a lack of sodium uptake apically would not compensate for the fact that there is not only a lack of chloride uptake but also active secretion through CFTR. In the interpretation of our findings on pulmonary edema in meningococcal septicemia, we hypothesized a role of inhibition of the basolateral Na/K ATPase. Against a sole involvement of and inhibition of the Na/K ATPase is the fact that we did not find a reduced renal fractional potassium excretion in patients with septicemia induced pulmonary edema. To accommodate our *in vivo* findings one would have to hypothesize an interaction between channel systems: an inhibition of the basolateral Na/K ATPase would have to be accompanied by uninhibited basolateral potassium channel activity maintaining potassium balance. The inhibition of the Na/K ATPase would then lead to a reduced sodium uptake through the apical sodium channels. At the same time, a cytokine-mediated activation of the NKCC1 channel system would increase intracellular chloride concentrations which would facilitate a simultaneous cytokine triggered CFTR activation, which would increase apical chloride extrusion. A simultaneous cytokine-mediated downregulation of CFTR expression and function (3) could explain the fact that the sodium/chloride ratio was consistently more than one in our *in vivo* measurements. Such a reduction in CFTR expression at the cell membrane would have to apply to both absorptive and secretory areas of the lung unlike suggested by the authors. There is no evidence that these two areas of the lung have different responses to, e.g., the CFTR suppressing cytokine tumor necrosis factor. How could the modified model I suggest be confirmed? Exhaled breath condensate (EBC) gained from the outgoing tubing of ventilators in patients ventilated because of respiratory failure from sepsis-related pulmonary edema or pneumonia could be gained following standardized and internationally recognized methods (4, 5) and analyzed for chloride and sodium levels relating these levels to urea concentrations to correct for evaporation and dilution and correcting for salivary contamination by amylase measurement. The levels obtained could then be compared to levels in patients ventilated for reasons unrelated to inflammation (e.g., post-surgery) in a prospective case–control study. Previous investigations of EBC comparing patients with cystic fibrosis to normal controls found no difference in chloride levels (6).

This means that if an increase in chloride levels which should be of similar magnitude or lower than the corresponding sodium levels was detected, this should be indicative of an underlying activation of CFTR and NKCC1 with overall chloride release moderated by a suppression of CFTR expression and, hence, in its magnitude matched or exceeded by an activation of extrusion of sodium through apical ENaC. Application of the NKCC1 inhibitors frusemide or bumetanide to patients with increased EBC chloride levels could confirm the hypothesis of a contribution of chloride excretion as these drugs should then lead to a reduction of EBC chloride levels.

## Author Contributions

ME conceived the comment and wrote the final version of the manuscript. He gave the final approval of the version to be published and agreed to be accountable for all aspects of the work in ensuring that, analysis, or interpretation of data for the work, and agreed to be accountable for all aspects of the work in ensuring that questions related to the accuracy or integrity of any part of the work are appropriately investigated and resolved.

## Conflict of Interest Statement

The author declares that the research was conducted in the absence of any commercial or financial relationships that could be construed as a potential conflict of interest.

## References

[1] Weidenfeld S Kuebler WM Cytokine-regulation of Na+-K+-Cl- cotransporter 1 and cystic fibrosis transmembrane conductance regulator-potential role in pulmonary inflammation and edema formation. Front Immunol (2017) 8:393 10.3389/fimmu.2017.00393 28439270 PMC5383711

[2] Eisenhut M Wallace H Barton P Gaillard E Newland P Diver M Pulmonary edema in meningococcal septicemia associated with reduced epithelial chloride transport. Pediatr Crit Care Med (2006) 7(2):119–24. 10.1097/00130478-200603000-00032 16446600

[3] Nakamura H Yoshimura K Bajocchi G Trapnell BC Pavirani A Crystal RG Tumor necrosis factor modulation of expression of the cystic fibrosis transmembrane conductance regulator gene. FEBS Lett (1992) 314(3):366–70. 10.1016/0014-5793(92)81507-I 1281791

[4] Carter SR Davis CS Kovacs EJ. Exhaled breath condensate collection in the mechanically ventilated patient. Respir Med (2012) 106(5):601–13. 10.1016/j.rmed.2012.02.003 22398157 PMC3314159

[5] Horváth I Hunt J Barnes PJ Alving K Antczak A Baraldi E Exhaled breath condensate: methodological recommendations and unresolved questions. Eur Respir J (2005) 26:523–48. 10.1183/09031936.05.00029705 16135737

[6] Zacharasiewicz A Wilson N Lex C Li A Kemp M Donovan J Repeatability of sodium and chloride in exhaled breath condensates. Pediatr Pulmonol (2004) 37:273–5. 10.1002/ppul.10431 14966822

